# Supplementary material for: An Evolutionary Paradigm Favoring Cross Talk between Bacterial Two-Component Signaling Systems
Source: mSystems. 2022 Oct 20;7(6):e00298-22. doi: 10.1128/msystems.00298-22 (PMC9765234; doi:10.1128/msystems.00298-22)
Supplement: TEXT S1 [file msystems.00298-22-s0001.docx]

# TEXT S1. Alternative fitness formulation and K_A_/K_S_ analysis

# Alternative fitness formulation

To test the robustness of our predictions to the details of the fitness formulation, we constructed an alternative fitness function and evaluated its implications:

In the absence of the input signal *I_i_*, the fitness is the maximum, i.e., one. The fitness decreases with increase in *I_i_* in a saturable manner with the half-saturation concentration *I_m_*. The fitness recovers with the response, which is proportional to the fraction of free promoters $f_{f}$. The expression for $f_{f}$ derived earlier (Eq. (18) in Methods) is employed above. This alternative formulation is used to estimate the fitness of various phenotypes as in Fig. 1. The selection coefficients of all the phenotypes for N=2 in programmed and random environments, respectively, estimated for several values of γ are in Fig. S2a and Fig. S2b. Phenotype 2, with one-way crosstalk mirroring the signal sequence, had the highest fitness in a programmed environment, whereas phenotype 1, without any crosstalk, had the highest fitness in a random environment, indicating the robustness of our results to the fitness formulation.

# K_A_/K_S_ analysis

Previous studies have argued that strong evolutionary pressure drives the diversification of TCS genes to ensure specificity following the creation of a new TCS by gene duplication (1, 2). We hypothesized that this pressure must be substantially lower, if at all, for TCSs between which crosstalk would be favored because of adaptation to a programmed environment. To test this hypothesis, following previous studies (2), we analyzed the sequences of the TCS genes of *M. tuberculosis* and estimated the ratio of non-synonymous to synonymous mutations (K_A_/K_S_). K_A_/K_S_ analysis has been employed previously to analyze the propensity of crosstalk between TCSs (2). Here, K_S_ is the number of synonymous mutations per synonymous site and K_A_ is the number of non-synonymous mutations per non-synonymous site. In general, if K_A_/K_S_<1, then selection pressure is thought to conserve protein sequences, whereas if K_A_/K_S_>1, the pressure is to diversify the genes (2, 3). For crosstalk, the binding regions of the noncognate HK and RR pairs must not be signiﬁcantly diﬀerent from those of their corresponding cognate pairs. Evolutionary pressure must therefore act to conserve these sequences post gene duplication. In other words, K_A_/K_S_ for TCSs that crosstalk must be smaller than for specific TCSs.

We downloaded both the nucleotide and amino acid sequences of the TCSs of *M. tuberculosis* from the National Center for Biotechnology Information’s (NCBI) GenBank database (4). We identified the kinase domain of each HK and the receiver domain of each RR using InterPro (5). These are the domains involved in the binding of HKs with RRs, required for phosphotransfer. We next aligned the nucleotide sequences of the domains, guided by their corresponding amino acid alignments, using the online tool Clustal Omega (6) (Fig. S6a, Fig. S6b) and evaluated the sequence similarity of the HK and RR domains (Fig. S6c, Fig. S6d).

Next, we identified TCSs for which K_A_/K_S_ could be estimated. Entities that share ancestry in gene duplication events have the possibility of crosstalk (7). Using whole protein amino acid sequences, we constructed phylogenetic trees separately for HKs (Fig. S7a) and RRs (Fig. S7b) following the maximum likelihood method with 500 bootstrap replicates using the MEGA (version 7) software package (8). We aligned the domain sequences of HKs and RRs separately. We considered HK-RR pairs connected within two levels of ancestry for K_A_/K_S_ estimation. We assumed that the corresponding TCSs had arisen from gene duplication events. For TCSs farther away in the trees, acquisition by horizontal gene transfer is more difficult to rule out (7). The K_A_/K_S_ ratios were estimated using the MEGA (version 7) software package (8). We calculated *p* values using a one-tailed paired *t*-test.

TCSs of *M. tuberculosis* have been classiﬁed into two major groups based on their homology (9). The first group, the NarL family, consists of Nar, Dev, and Pdta TCSs. The second group, the OmpR family, contains the rest. Our trees were consistent with this classiﬁcation. (We neglected the atypical TCS Rv0600c-Rv0601c-TcrA, which does not undergo autophosphorylation (10).) From the trees, we identified HKs and RRs connected within two levels of ancestry. This yielded the following related HKs (Fig. S7a): 1) NarS and DevS/DosT (green node); 2) MprB, PrrB, and SenX3 (blue node); and 3) PhoR, TrcS, and TcrY (yellow node). Similarly, the following related RRs emerged (Fig. S7b): 1) PdtaR, NarL, and DevR (green node); 2) MprA, PrrA, and KdpE (blue node); and 3) PhoP, TrcR, and TcrX (yellow node). Note that the HKs and RRs in nodes of the same color belong largely to the same TCSs. For instance, the yellow node has the PhoR-PhoP, TrcS-TrcR, and TcrY-TcrX TCSs in both the trees. This gave us further confidence in their possible connectedness through gene duplication. The kinase domain has not been annotated for the NarS gene (5). We thus could not analyze the green node. We analyzed sequence data from the other two nodes.

We recall from the *in vitro* data (Main text, Fig. 4a) that in the blue nodes, PhoR-PhoP and TcrY-TcrX crosstalk, whereas, in the yellow nodes, MprB-MprA and PrrB-PrrA crosstalk. The other pairs do not. We estimated the K_A_/K_S_ ratios for all the HK pairs and the RR pairs in the yellow and blue nodes (Fig. S7c, Table S2). The ratios were 0.59±0.18 for the HKs and 0.57±0.09 for the RRs, indicating no significant overall difference in evolution between the HKs and RRs. Because the K_A_/K_S_ ratios can vary between the nodes, to assess the differences between the K_A_/K_S_ ratios of TCSs engaged in crosstalk and not, we performed the one-tailed paired *t*-test (paired by their nodes in the phylogenetic tree, since the genes are assumed to have been duplicated) for the HKs and RRs separately. Interestingly, we found that the ratios for the RRs engaged in crosstalk (PrrA-MprA and TcrX-PhoP) were signiﬁcantly lower than for those that did not (*p*=0.035). The difference was not significant for the HKs (*p*=0.433). The implication is that the binding domains in the RRs that crosstalk were under much less evolutionary pressure to diversify than those that were specific. The HKs were not under similar pressure. Note that crosstalk is possible if either the HKs or the RRs have their binding domains conserved. It is only if both diversify that speciﬁcity results. For instance, if HK_1_ is similar to HK_2_ but RR_1_ is removed from RR_2_, HK_1_ will still be able to crosstalk to RR_2_, because HK_2_ will continue to exhibit its cognate interaction with RR_2_. HK_1_ and HK_2_ must also become dissimilar for the two TCSs to become insulated. We may infer thus that TCSs that crosstalk may be under less evolutionary pressure to diversify post gene duplication than TCSs that are specific.

REFERENCES

1. Capra EJ, Laub MT. 2012. Evolution of two-component signal transduction systems. Annu Rev Microbiol 66:325-47.

2. Rowland MA, Deeds EJ. 2014. Crosstalk and the evolution of specificity in two-component signaling. Proc Natl Acad Sci U S A 111:5550-5.

3. Hurst LD. 2002. The Ka/Ks ratio: diagnosing the form of sequence evolution. Trends Genet 18:486.

4. Sayers EW, Cavanaugh M, Clark K, Ostell J, Pruitt KD, Karsch-Mizrachi I. 2020. GenBank. Nucleic Acids Res 48:D84-D86.

5. Mitchell A, Chang HY, Daugherty L, Fraser M, Hunter S, Lopez R, McAnulla C, McMenamin C, Nuka G, Pesseat S, Sangrador-Vegas A, Scheremetjew M, Rato C, Yong SY, Bateman A, Punta M, Attwood TK, Sigrist CJ, Redaschi N, Rivoire C, Xenarios I, Kahn D, Guyot D, Bork P, Letunic I, Gough J, Oates M, Haft D, Huang H, Natale DA, Wu CH, Orengo C, Sillitoe I, Mi H, Thomas PD, Finn RD. 2015. The InterPro protein families database: the classification resource after 15 years. Nucleic Acids Res 43:D213-21.

6. McWilliam H, Li W, Uludag M, Squizzato S, Park YM, Buso N, Cowley AP, Lopez R. 2013. Analysis tool web services from the EMBL-EBI. Nucleic Acids Res 41:W597-600.

7. Alm E, Huang K, Arkin A. 2006. The evolution of two-component systems in bacteria reveals different strategies for niche adaptation. PLoS Comput Biol 2:e143.

8. Kumar S, Stecher G, Tamura K. 2016. MEGA7: molecular evolutionary genetics analysis version 7.0 for bigger datasets. Mol Biol Evol 33:1870-4.

9. Tyagi JS, Sharma D. 2004. Signal transduction systems of mycobacteria with special reference to *M. tuberculosis*. Current Science:93-102.

10. Bretl DJ, Demetriadou C, Zahrt TC. 2011. Adaptation to environmental stimuli within the host: two-component signal transduction systems of *Mycobacterium tuberculosis*. Microbiol Mol Biol Rev 75:566-82.
