# Supplementary figures and images for: An Evolutionary Paradigm Favoring Cross Talk between Bacterial Two-Component Signaling Systems
Source: mSystems. 2022 Oct 20;7(6):e00298-22. doi: 10.1128/msystems.00298-22 (PMC9765234; doi:10.1128/msystems.00298-22)

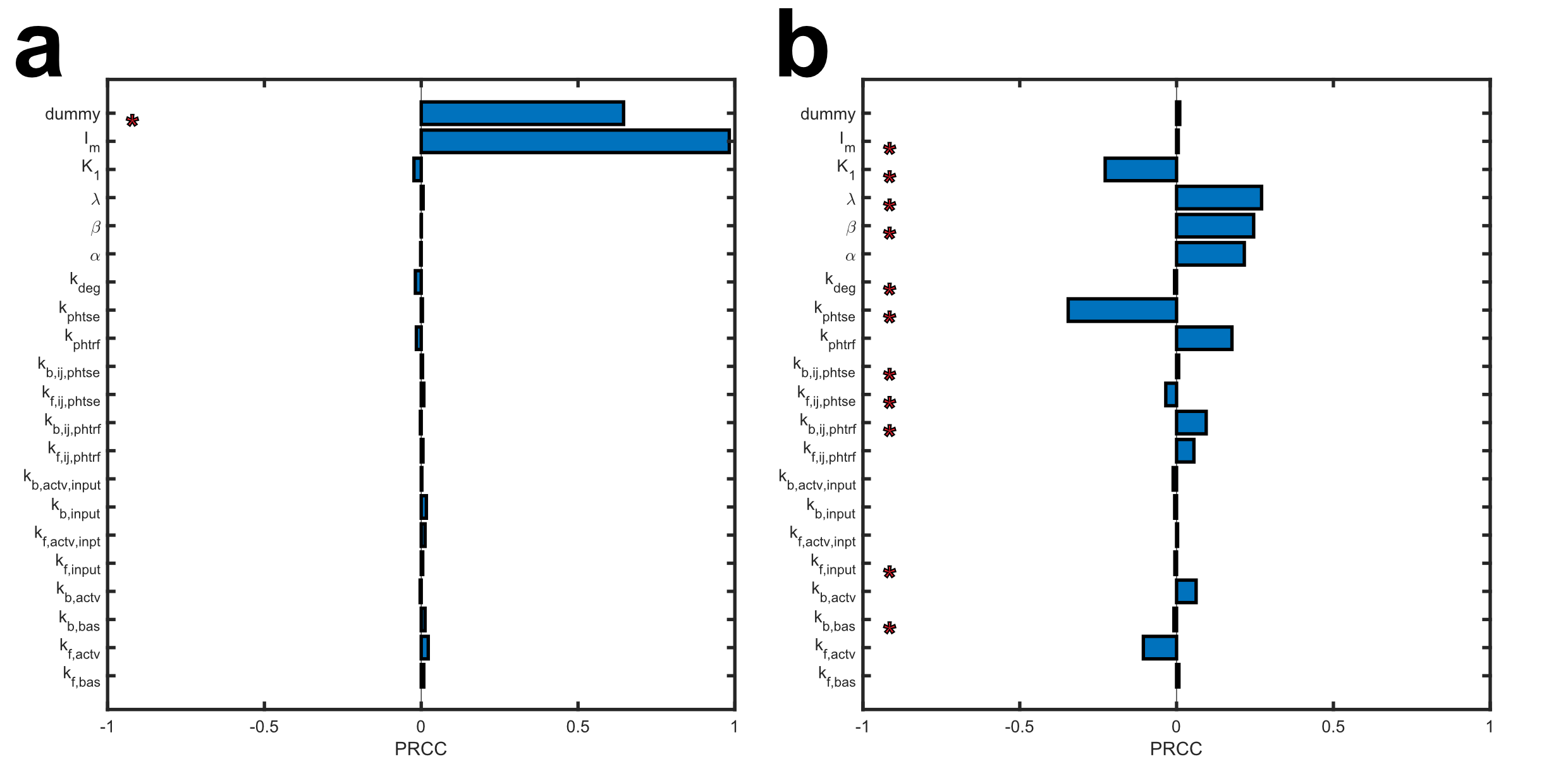

Supplement: FIG S1 [file msystems.00298-22-s0004.tif]

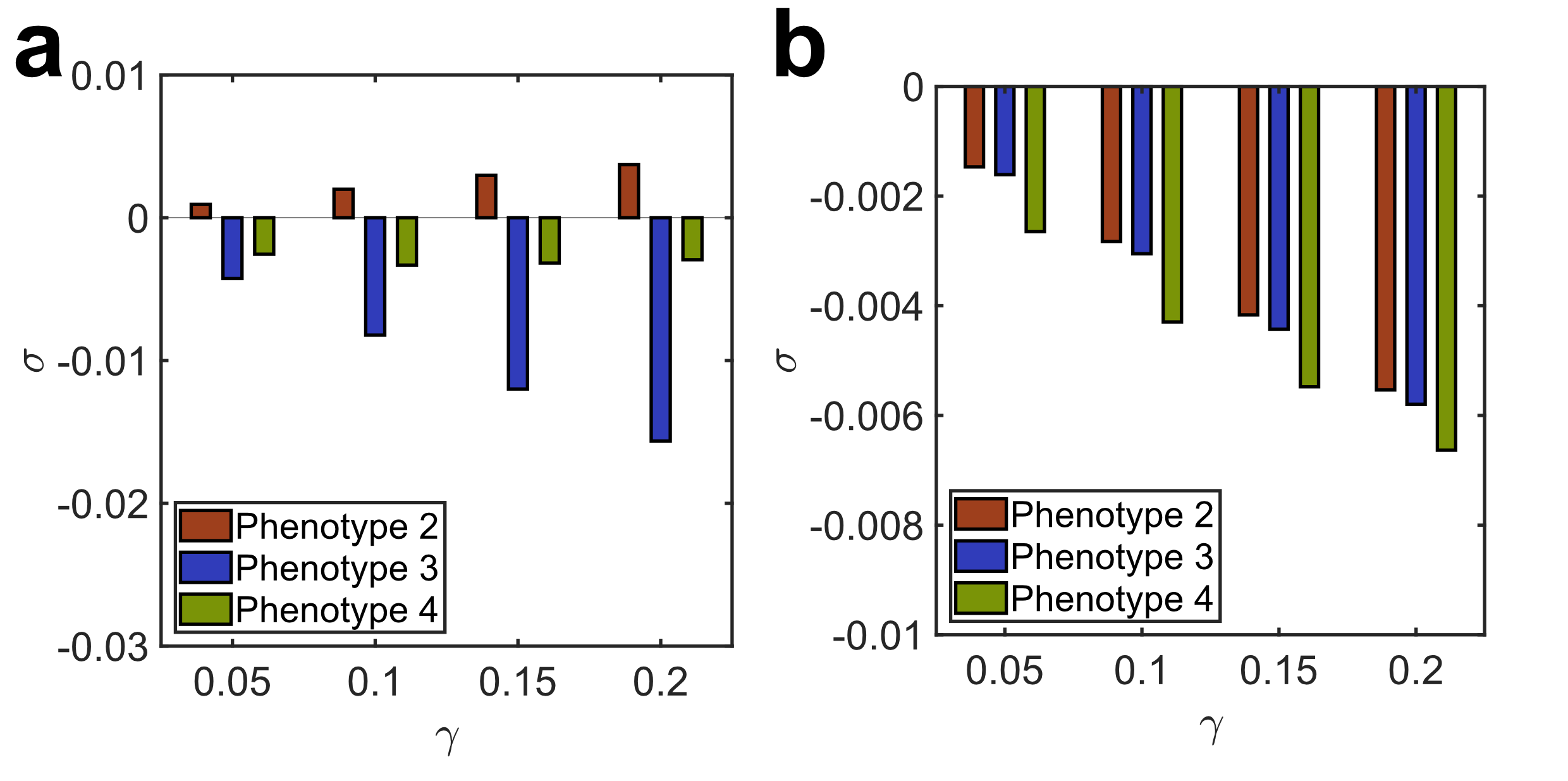

Supplement: FIG S2 [file msystems.00298-22-s0005.tif]

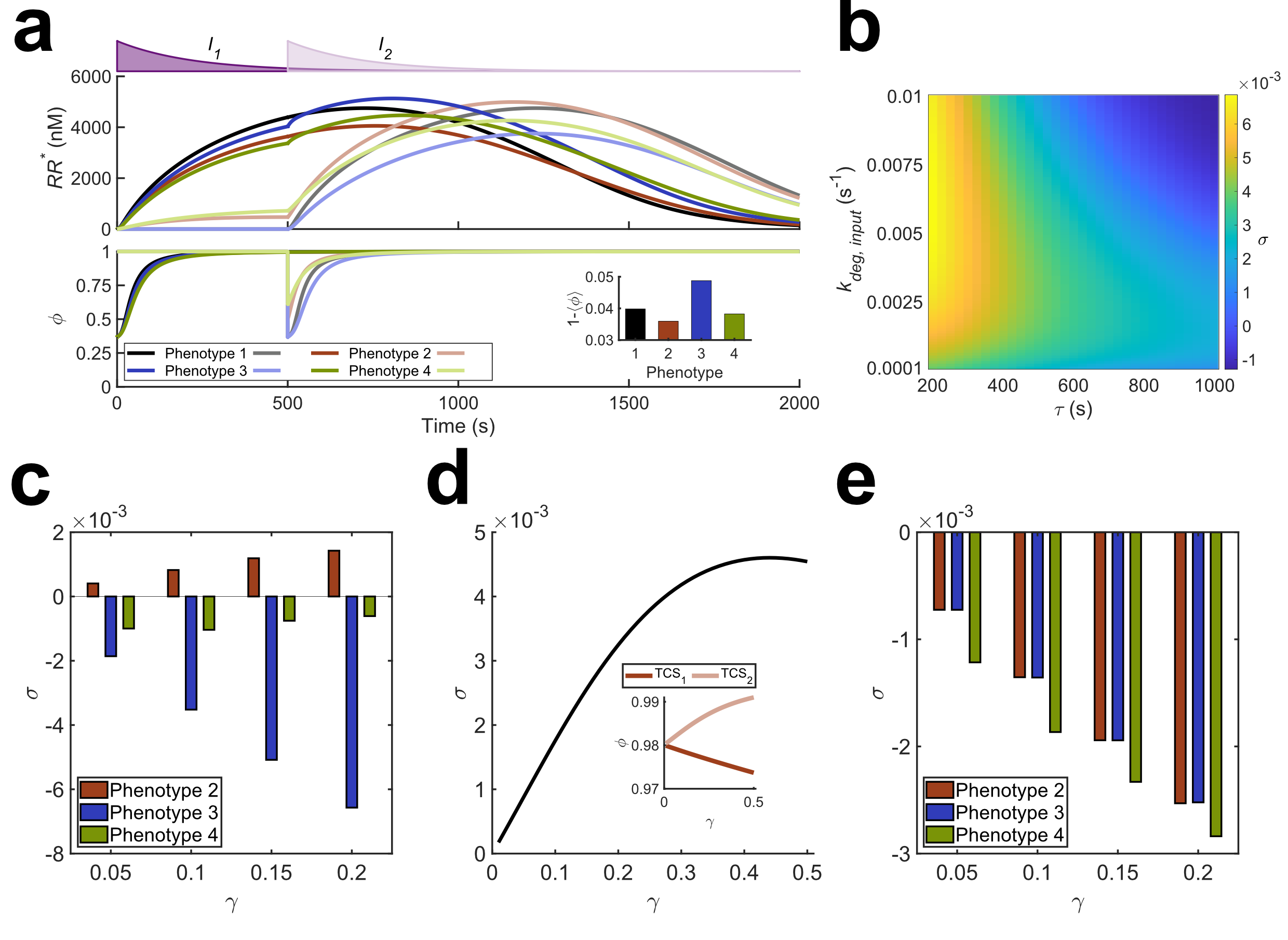

Supplement: FIG S3 [file msystems.00298-22-s0006.tif]

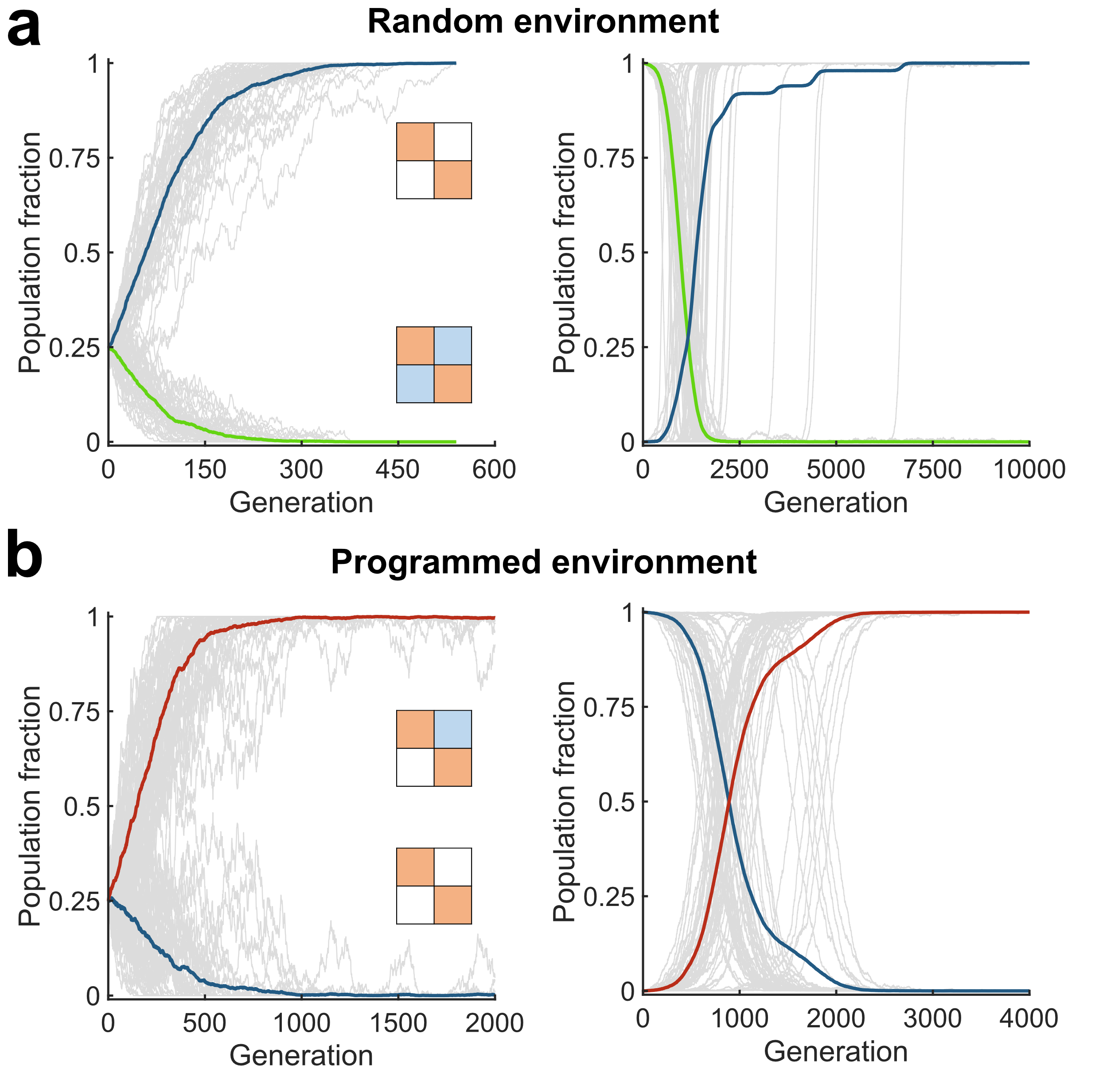

Supplement: FIG S4 [file msystems.00298-22-s0007.tif]

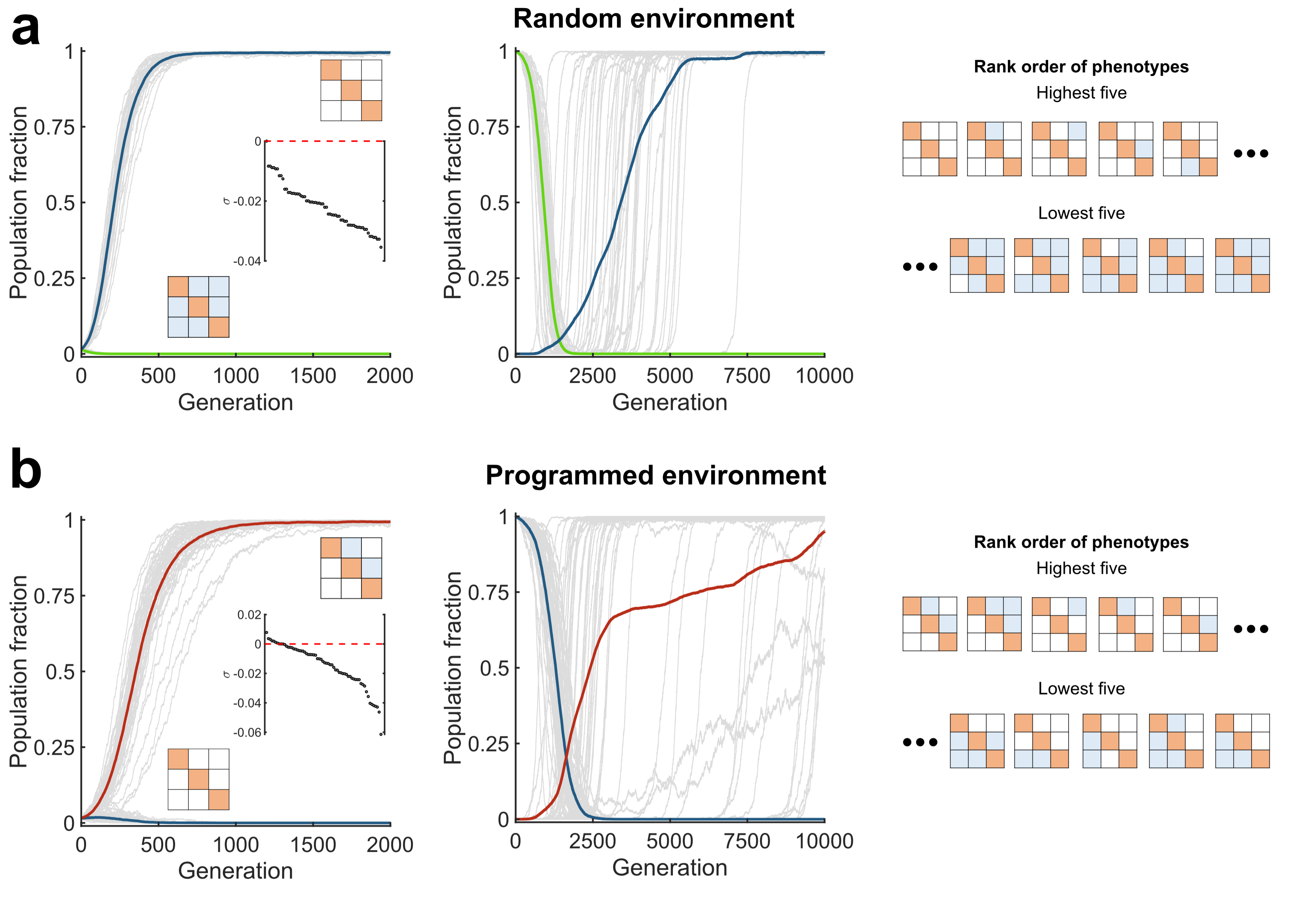

Supplement: FIG S5 [file msystems.00298-22-s0008.tif]

*pdtaS*/1-68  
*devS*/1-62  
*dosT*/1-61  
*prpB*/1-62  
*mprB*/1-72  
*kdpD*/1-68  
*senX3*/1-66  
*mtrB*/1-67  
*tcrY*/1-65  
*trcS*/1-67  
*phoR*/1-63

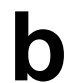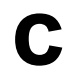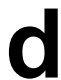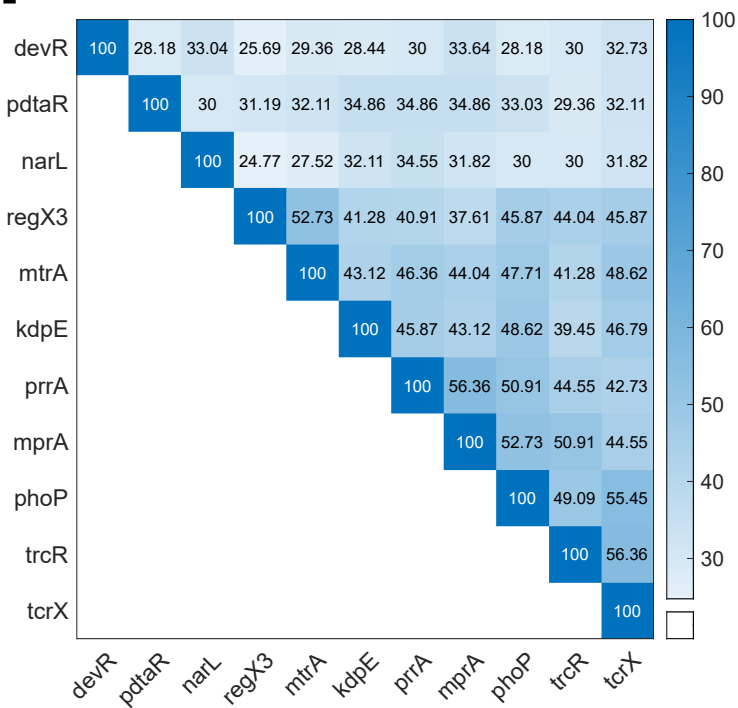

Supplement: FIG S6 [file msystems.00298-22-s0009.pdf]

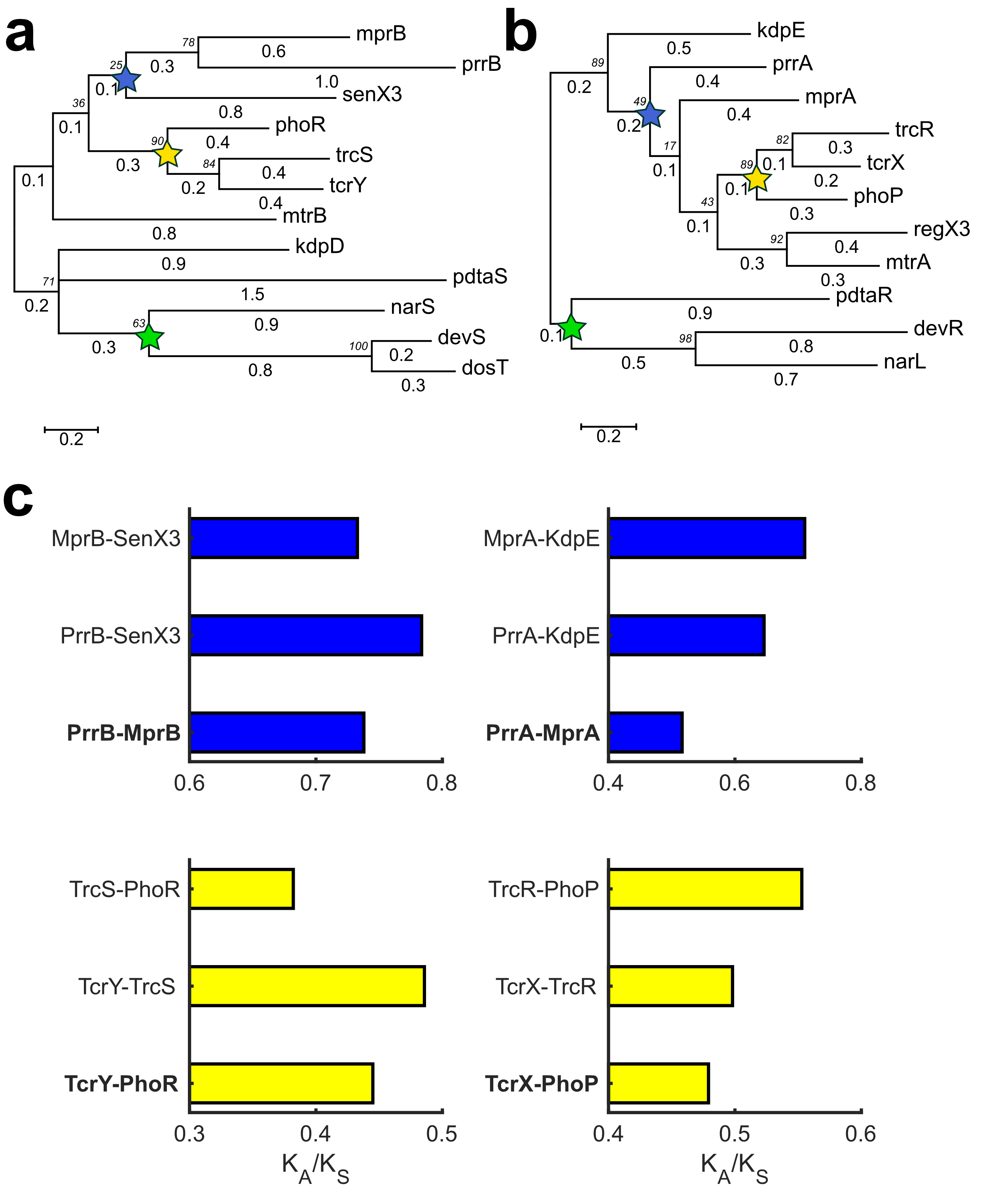

Supplement: FIG S7 [file msystems.00298-22-s0010.tif]
