# Supplementary material for: An Evolutionary Paradigm Favoring Cross Talk between Bacterial Two-Component Signaling Systems
Source: mSystems. 2022 Oct 20;7(6):e00298-22. doi: 10.1128/msystems.00298-22 (PMC9765234; doi:10.1128/msystems.00298-22)
Supplement: TABLE S2 [file msystems.00298-22-s0003.docx]

**TABLE S2. K_A_/K_S_ ratios.** The K_A_/K_S_ ratios for the HKs and RRs identified in the colored nodes of the phylogenetic trees in Fig. S7. (Also see text, Fig. 4a).

| Node | *HK_1_* | *HK_2_* | K_A_/K_S_ | *RR_1_* | *RR_2_* | K_A_/K_S_ |
| --- | --- | --- | --- | --- | --- | --- |
| **Blue** | PrrB | MprB | 0.7379 | PrrA | MprA | 0.5165 |
|  | PrrB | SenX3 | 0.7836 | PrrA | KdpE | 0.6461 |
|  | MprB | SenX3 | 0.7328 | MprA | KdpE | 0.7104 |
| **Yellow** | TcrY | PhoR | 0.4450 | TcrX | PhoP | 0.4791 |
|  | TcrY | TrcS | 0.4858 | TcrX | TrcR | 0.4981 |
|  | TrcS | PhoR | 0.3821 | TrcR | PhoP | 0.5529 |
