# Supplementary material for: An Evolutionary Paradigm Favoring Cross Talk between Bacterial Two-Component Signaling Systems
Source: mSystems. 2022 Oct 20;7(6):e00298-22. doi: 10.1128/msystems.00298-22 (PMC9765234; doi:10.1128/msystems.00298-22)
Supplement: TABLE S1 [file msystems.00298-22-s0002.docx]

**TABLE S1. Model parameters and initial conditions.**

| Symbol | Description | Value | Source |
| --- | --- | --- | --- |
| $P_{T}$ | Total promoter concentration | 100 nM | (1) |
| $k_{f,bas}$ | Basal autophosphorylation rate | 10^-10^ s^-1^ | (1) |
| $k_{f,actv}$ | $I_{i}HK_{i}$ autophosphorylation rate | 0.1 s^-1^ | (1) |
| $k_{b,bas}$ | Basal dephosphorylation rate | 0.1 s^-1^ | (1) |
| $k_{b,actv}$ | $I_{i}HK_{i}^{*}$ dephosphorylation rate | 0.1 s^-1^ | (1) |
| $k_{f,input}$ | $I_{i}-HK_{i}$ binding rate | 100 nM^-1^s^-1^ | (1) |
| $k_{f,actv,input}$ | $I_{i}-HK_{i}^{*}$ binding rate | 100 nM^-1^s^-1^ | (1) |
| $k_{b,input}$ | $I_{i}HK_{i}$ unbinding rate | 1000 s^-1^ | (1) |
| $k_{b,actv,input}$ | $I_{i}HK_{i}^{*}$ unbinding rate | 10^-6^ s^-1^ | (1) |
| $k_{f,ij,phtrf}$ | $HK_{i}^{*}-RR_{j}$ binding rate | γ×10^-3^ nM^-1^s^-1^ | (1) |
| $k_{b,ij,phtrf}$ | $HK_{i}^{*}-RR_{j}$ unbinding rate, $\forall i,j$ | 1 s^-1^ | (1) |
| $k_{f,ij,phtse}$ | $HK_{i}-RR_{j}^{*}$ binding rate | γ×10^-3^ nM^-1^s^-1^ | (1) |
| $k_{b,ij,phtse}$ | $HK_{i}-RR_{j}^{*}$ unbinding rate, $\forall i,j$ | 1 s^-1^ | (1) |
| $k_{phtrf}$ | Phosphotransfer rate | 0.055 s^-1^ | - |
| $k_{phtse}$ | Cognate phosphatase activity rate | 0.01 s^-1^ | - |
|  | Non-cognate phosphatase activity rate | 1.67×10^-3^ s^-1^ |  |
| $k_{deg}$ | Protein degradation rate | 6×10^-5^ s^-1^ | (2) |
| $\alpha$ | Ratio of basal and activated transcription rates | 10 | (2) |
| $\beta$ | $HK-RR$ synthesis rate | 6×10^-3^ nM s^-1^ | (2) |
| $\lambda$ | Ratio of $HK$ and $RR$ concentrations | 0.1 | (2) |
| $K_{1}$ | Dissociation constant for $(RR_{j}^{*})^{2}P_{j}$ | 5×10^5^ nM^2^ | (2) |
| $\mu$ | Mutation rate | 10^-5^ per crosstalk interaction, per generation | Assumed |
| $k_{deg,input}$ | Input degradation rate | 4.605×10^-3^ s^-1^ (for exponentially decaying input) | Assumed |
| $\gamma$ | Fold change in $k_{f,ij,phtrf}$ and $k_{f,ij,phtse}$ | 1 if $i=j$, else varied | - |
| $HK(0)$ | Initial concentration of HKs | 100 nM | (1) |
| __ | Initial concentration of RRs | 1000 nM | (1) |
| $I_{m}$ | Peak stimulus strength | 10^4^ nM | (1) |

The abbreviations used in the subscripts of the parameter symbols are:

*f*: forward; *b*: backward; *bas*: basal; *actv*: activated; *phtrf*: phosphotransfer; *phtse*: phosphatase; *deg*: degradation

**REFERENCES**

1. Rowland MA, Deeds EJ. 2014. Crosstalk and the evolution of specificity in two-component signaling. Proc Natl Acad Sci U S A 111:5550-5.

2. Tiwari A, Balazsi G, Gennaro ML, Igoshin OA. 2010. The interplay of multiple feedback loops with post-translational kinetics results in bistability of mycobacterial stress response. Phys Biol 7:036005.
